# Peak Systolic Velocity of Superior Thyroid Artery for the Differential Diagnosis of Thyrotoxicosis

**DOI:** 10.1371/journal.pone.0050051

**Published:** 2012-11-16

**Authors:** Xiaolong Zhao, Lili Chen, Ling Li, Yao Wang, Yong Wang, Linuo Zhou, Fangfang Zeng, Yiming Li, Renming Hu, Hong Liu

**Affiliations:** 1 Department of Endocrinology, Huashan Hospital, Shanghai Medical College, Fudan University, Shanghai, China; 2 Department of Endocrinology, Zhongda Hospital of Southeast University, Nanjing, China; 3 Department of Ultrasound, Huashan Hospital, Shanghai Medical College, Fudan University, Shanghai, China; Cardiff University, United Kingdom

## Abstract

**Aim:**

The differentiation of destruction-induced thyrotoxicosis and Graves’ disease (GD) is of great importance for selection of proper therapy. Radioactive iodine uptake (RAIU) is the gold standard for differentiating these two conditions but its application has remained somewhat limited. Thyroid color Doppler flow sonography (CDFS) is a potential alternative of RAIU but more supporting evidence is warranted. In the present study, a standard operative procedure was developed to measure the mean peak systolic velocity of superior thyroid artery (STA-PSV) and evaluate its role in the differential diagnosis of thyrotoxicosis.

**Methods:**

A total of 135 patients with untreated thyrotoxicosis were enrolled into one retrospective study (GD, n = 103; thyroiditis, n = 32) and another prospective study recruited 169 patients (GD, n = 118; thyroiditis, n = 51). Thirty normal controls were also enrolled. Thyroid function, anti-TSH-receptor antibody (TRAb), RAIU, CFDS of thyroid and STA-PSV were performed for each patient. Receiver operator curve (ROC) was used to evaluate the diagnostic value of STA-PSV in a retrospective study so as to seek the optimal cutoff point. Then the cutoff point value was used to validate its diagnostic value in a prospective study and in another thyrotoxicosis population.

**Results:**

STA-PSV of GD was significantly higher than that of thyroiditis in both retrospective and prospective studies. The area under the ROC curve of mean STA-PSV was 0.8799 and 0.9447 in the retrospective and prospective studies respectively. If a mean STA-PSV cutoff point of 50.5 cm/s was set from the retrospective analysis for the prospective study, the sensitivity and specificity in distinguishing GD from thyroiditis were 81.04% and 96.08% respectively. Mean STA-PSV and TRAb had similar area under ROC. The coefficients of variation in STA-PSV measurement were lower than 10% for the euthyroid, thyroiditis and GD groups.

**Conclusions:**

STA-PSV is a feasible supplement alternative of RAIU for differentiating the causes of thyrotoxicosis.

## Introduction

As mentioned in the 2011 ATA (American Thyroid Association) guideline, the color Doppler flow sonography (CDFS) of thyroid, as an indicator of thyroid blood flow status, has some practical values in the differentiation of thyrotoxicosis. However, due to lack of well-designed and evidence-based diagnostic trials, official recommendation is not made. It is only limited to the differentiation of some special circumstances, such as amiodarone-induced thyroiditis [1]. From the perspectives of clinical trial design, the previous studies were mainly descriptive and were lack of ROC curve analysis [2], [3]. And there was no validation of the study conclusions among another population [2], [3], [4]. Furthermore, reproducibility analysis was not performed for the measurement. The study sample was relatively small [2], [3], [4]. Some studies evaluated the thyroid blood flow pattern with CDFS [4]. However, the rough estimate of blood flow mode is very subjective and difficult for quantification. Moreover, the quantification of thyroid blood supply required complicated post-examination software processing [4]. From the perspective of blood vessel detection, it is rather difficult to accurately position while measuring the velocity of inferior thyroid artery [2]. Also, the variation of vessel lumen sizes and artery spreading directions may interfere with the blood flow velocity detection [5]. The inferior thyroid artery is commonly used in clinical practice [3]. However, since it is deeply located [6], accurate positioning is difficult. By contrast, STA is superficial, and can be easily positioned from its intersecting point with thyroid lobes. In addition, the anatomic variation of STA rarely occurs [7]. Hence, we chose the peak systolic velocity (PSV) of STA as the ideal marker of thyroid blood flow status.

The etiological differentiation of thyrotoxicosis is a commonly encountered issue in clinical practice. It is more complex for the diffuse toxic goiter, including destructive thyroiditis resulting from various causes and Graves’ disease (GD). The outcomes and treatment of these two conditions are different. For thyroiditis, symptomatic treatment is enough, since it is self-limiting. However, for GD, anti-thyroid drugs, surgery or isotope therapy is necessary. In recent years, with the wider popularity of physical health screening, more and more cases of atypical GD have been identified. There are no typical clinical symptoms, such as diffuse goiter, exophthalmus or pretibial myxedema. At the same time, the incidence of painless thyroiditis is rising. In this case, a rapid, convenient and dependable method for differentiating the causes of thyrotoxicosis is urgently needed. In clinical practice, radioactive iodine uptake (RAIU) is still the gold standard. However, it is not generally available and accessible in China. It is time-consuming and hard to operate, and can be influenced by iodine-containing diet and medications.

As an available, easily accessible, convenient and relatively accurate assay, CDFS is a good alternative. Based on the standard operating procedures (SOP) for measuring the PSV of STA, we employed retrospective studies to assess its differential diagnostic value in thyrotoxicosis. Then, prospective studies were used to validate the differential diagnostic value of STA-PSV. Furthermore, its diagnostic value was compared with those of TSH receptor antibody (TRAb) and FT3/FT4. In addition, the reproducibility of STA-PSV assay was measured. Finally, the optimal cutoff point of STA-PSV obtained from the above study was applied in hospitals adopting this technique as well as SOP, to validate if it is worthy of promotion. The present study is aimed to provide evidence and guidance for clinical practice.

**Table 1:** General characteristics of the study participants.

	Normal controls	Retrospective study		Prospective study	
		GD	Thyroiditis	GD	Thyroiditis
*n*	30	103	32	118	51
Male/female	11/19	29/74	12/20	42/76	18/33
Age (y)	40.1±11.2	45.8±13.6	43.0±12.3	39.6±13.7	45.0±12.1
FT3 (pM)	4.92±1.25	18.15±7.61	8.45±3.94* &	12.11(7.82–22.5)	8.53(5.12–12.64)* &
FT4 (pM)	20.12±5.42	58.57±36.82	29.62±15.82* &	38.1(26.9–59.8)	29.94(22.17–38.00)* &
TSH (mIU/L)	2.6(0.46–3.4)	0.11(0.0–0.16)	0.11(0.02–0.24)&	0.01(0.005–0.021)	0.025(0.008–0.200) &
Left STA-PSV	32.6±8.5	69.5±37.5	41.7±26.4* &	67.0(54.2–93.0)	30.0(25.0–40.0)* &
Right STA-PSV	30.2±7.4	70.7±30.8	35.9±18.1*	70(53.8–93.3)	34(27.0–41.9)* &
Mean STA-PSV	32.95±4.57	70.1±32.6	38.8±21.5*	67.93(56.0–95.7)	33(27–40)* &
3 h RAIU	N/A	52.72(35.2–88.4)	3.0(0.2–4.95)*	39.2(27.1–56.3)	2.30(1.21–3.21)*
24 h RAIU	N/A	67.0(60.8–79.5)	1.35(0.50–20.25)*	64.1(52.6–76.6)	3.3(0.32–7.82)
TPOAb	30(4.5–51)	N/A	N/A	53.7(30.0–196.8)	45(13.4–210.0)
TRAb	0.5(0–4)	N/A	N/A	18.9(7.27–31.25)	1.0(0.45–7.82)

* *P*<0.05 versus GD in retrospective and prospective studies;

& *P*<0.05 versus normal controls in retrospective and prospective studies;

N/A: Not available.

## Materials and Methods

### Ethics Statement

The ethics committee of Huashan Hospital and Zhongda Hospital approved the study protocol. All participants signed written informed consent.

**Figure 1:**
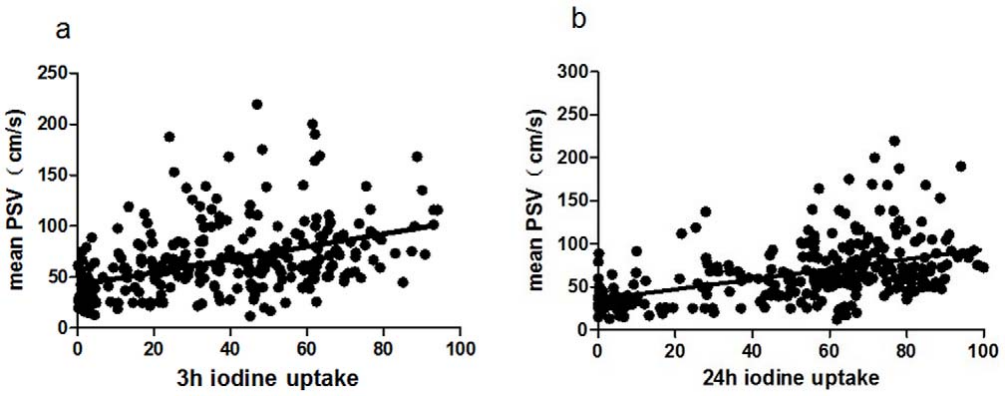
Correlations of mean STA-PSV with 3 h and 24 h RAIU.

### Patients

For the retrospective analysis, a total of 135 patients with thyrotoxicosis were enrolled in Huashan Hospital of Fudan University from February 2006 to June 2009. All participants underwent RAIU, STA-PSV and thyroid function measurements altogether, in less than 1 week. Diffuse goiter with autonomous functional or multiple nodules were excluded by sonography. RAIU test was set as the gold standard. Appropriate reference range was used, as recommended by the study on the normal range of RAIU after universal salt iodization in China [8]. Thyrotoxicosis is defined as elevated FT3, elevated FT4 and suppressed TSH. Thyrotoxicosis with suppressed RAIU (3 h<10% and 24 h<45%) was diagnosed as thyroiditis (*n* = 33). Thyrotoxicosis with elevated RAIU (3 h≥10% or 24 h≥45%) was diagnosed as GD (*n* = 102) [4]. Subacute thyroiditis was diagnosed in patients with fever, neck pain or swelling in the presence of thyroiditis. Thyroiditis without neck pain was diagnosed as painless thyroiditis. If painless thyroiditis occurred within 1 year postpartum, it was considered as postpartum thyroiditis [4].

**Figure 2:**
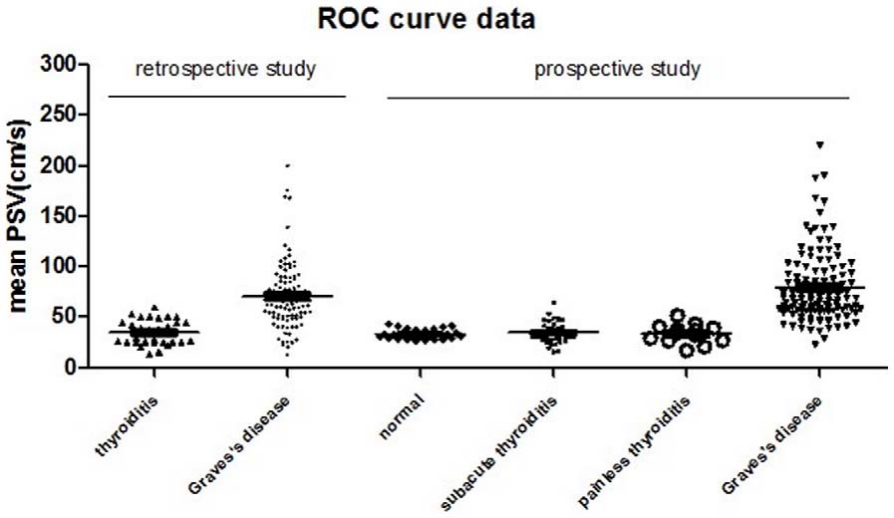
Distribution of mean STA-PSV of normal subjects and patients with thyroiditis and GD.

**Table 2:** Mean STA-PSV for the differential diagnosis of thyrotoxicosis.

	Retrospective study		Prospective study (Huashan Hospital)		Prospective study (Zhongda Hospital)	
	GD	Thyroiditis	GD	Thyroiditis	GD	Thyroiditis
Mean STA-PSV>50.5 cm/s	73	1	96	2	34	0
Mean STA-PSV< = 50.5 cm/s	30	31	22	49	1	5
Total	103	32	118	51	35	5
Sensitivity	70.87%		81.36%		97.14%	
Specificity	96.88%		96.08%		100%	
Acc	77.0%		85.8%		97.5%	
+PV	98.6%		98.0%		100%	
−PV	50.8%		69.1%		83.3%	
+LR	22.68		20.75		∞	

ACC: Accuracy.

+PV: Positive predictive value.

−PV: Negative predictive value.

+LR: Positive likelihood ratio.

Simultaneously, a prospective study (clinical trial: NCT01227499) was conducted to validate the efficacy of this diagnosis test from July 2009 to November 2011 at two clinical centers (Huashan Hospital of Fudan University and Zhongda Hospital of Southeast University) from July 2009 to November 2011. The endocrinologists and ultrasonologists in Zhongda Hospital had never performed this examination before, and they carried it out according to our SOP. The inclusion criterion was newly-diagnosed thyrotoxicosis without antithyroid intervention, including antithyroid drugs (ATD) and β–blockers. The diagnosis of GD and thyroiditis was the same as above. If the laboratory test and RAIU were inconsistent with the clinical impressions, the patient would be followed up for 3 months to confirm the diagnosis [4]. The exclusion criteria were multinodular goiter; toxic nodular goiter; history of thyroid surgery, radioactive iodine treatment, or neck irradiation; medications such as ATDs or thyroxine pills taken in 2–4 weeks; foods with high iodine content taken in 2–4 weeks, such as sea tangle or laver; iodine-containing medications such as contrast medium or amiodarone; pregnancy or lactation.

At the same time, thirty normal and healthy (normal thyroid function, negative TPO antibody and normal thyroid morphology on color Doppler), age and gender-matched participants were recruited as normal controls.

**Figure 3:**
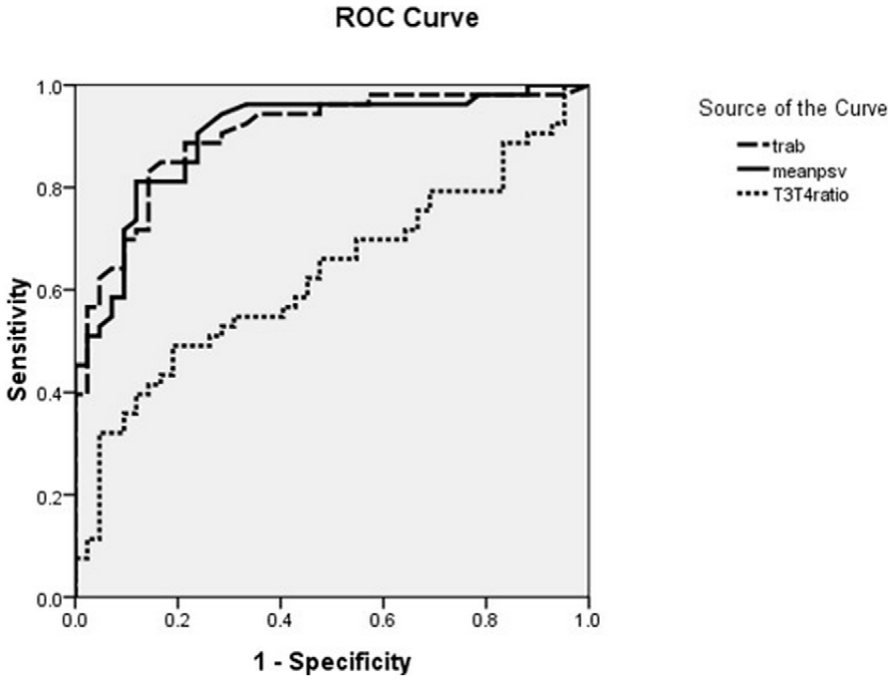
Capacity for the differentiation of thyrotoxicosis: TRAb, FT3/FT4, and mean STA-PSV.

### Measurement of Serological Makers

Blood samples were collected from all study participants. The serum concentrations of FT4, FT3 and TSH were measured by electrochemiluminescence immunoassay (Elecsys E170, Roche Diagnostics, Tokyo, Japan) with an intra-assay coefficient of variation (CV) of 3.9% and an interassay CV of 8.9%. TRAb was measured by enzyme-linked immunosorbent assay (ELISA; ElisaRSR™ TRAb 3rd generation kit; RSR, Cardiff, UK) with an intra-assay CV of 4.2% and an inter-assay CV of 9.1%. Thyroid peroxidase antibody (TPOAb) and thyroglobulin antibody (TgAb) were measured by radioimmunoassay (RiaRSR™ TPOAb, RiaRSR™ TgAb; RSR, Cardiff, UK) with an intra-assay CV of 4.2% and an inter-assay CV of 6.8%. Normal values were as follows: FT4, 10.2–31.0 pmol/l; FT3, 3.5–6.5 pmol/l; TSH, 0.350–5.500 mIU/l; TgAb, 0 mIU/ml; TPOAb, 0 mIU/ml; and TRAb,<5 U/l.

**Table 3:** Coefficient of variation of the STA-PSV detection.

	Euthyroid	Thyroiditis	Graves’ disease
Left STA-PSV (%)	8.35±1.28	9.56±1.42	7.58±1.47
Right STA-PSV (%)	6.40±1.24	5.28±1.36	3.96±1.23
Mean STA-PSV (%)	4.96±1.20	4.96±1.33	3.50±1.22

### Color Doppler Flow Sonography

Color Doppler flow sonography (CDFS) of thyroid gland was performed in all patients by one expert at each clinical center with ALOKA A10 Color Doppler at Huashan Hospital and GE LOGIQ 9 Color Doppler at Zhongda Hospital respectively. Imaging system in connection with a 7.5-MHZ linear transducer was provided by the manufacturer and followed with SOP. After a 10-minute rest, the patients were examined in supine position. Each lobe of thyroid was scanned longitudinally and transversally, in both B-mode and color-flow-mode. During the transversal scanning, the superior thyroid artery was pinpointed. The crossing point of superior thyroid artery and the edge of thyroid lobe was set as the positioning site. For PSV measurements, the sampling volume was adjusted to 3 mm at the center of vessel, the insonation angle maintained at 0°–60° and the angle of correction adjusted so that it was parallel to the direction of blood flow. Left STA was identified and the sample-volume cursor positioned within the axis of a straight stretch of vessel. The spectral wave form was stored digitally and the measurements were taken in triplicates to calculate the mean value. It was repeated for right STA. Then the average value of left STA-PSV and right STA-PSV was calculated as the mean STA-PSV. All performers were blind to the patient’s previous diagnosis.

Five euthyroid people, 5 patients with thyroiditis and 5 with GD were sampled randomly to detect the PSV of left and right STA. The measurements were repeated for 10 times to calculate the coefficient of variation.

**Table 4:** Accordance analysis between TRAb and PSV in the prospective study.

	Consistent	Inconsistent	
Thyroiditis (n = 51)	34	15 GD according to TRAb	2 GD according to PSV
GD (n = 118)	80	22 thyroiditis according to PSV	16 thyroiditis according to TRAb

GD, Graves’ disease.

mean-PSV>50.5 cm/s: GD; mean-PSV≤50.5 cm/s: thyroiditis.

TRAb<5 U/L: thyroiditis; TRAb≥5 U/L: GD.

### Statistical Analysis

The normally distributed data were expressed as means ± SD while the variables with a skewed distribution were reported as median (interquartile range). Student’s t or Mann-Whitney U test was used to compare the intra-group differences. The sensitivity, specificity and receiver operating characteristic (ROC) curves were calculated by a two-by-two contingency table. And the optimal cutoff point was yielded from the closest point to the left upper corner on the ROC curve. Then the optimal cut-off value of mean STA-PSV was used to judge its differential value in a prospective study. All statistical analyses were performed with the SPSS Statistical Package (version 13.0; SPSS Inc., Chicago, IL). Values of *P*<0.05 were considered statistically significant.

## Results

### Patient Characteristics

A total of 135 patients with thyrotoxicosis were enrolled into a retrospective study and 169 patients with thyrotoxicosis were enrolled into another prospective study. Both were performed in Huashan Hospital. In the prospective study, there were 118 GD patients, 5 patients with postpartum thyroiditis, 12 patients with painless thyroiditis and 34 patients with subacute thyroiditis. Table 1 summarized the clinical characteristics as well as the results of their thyroid function, RAIU and CDFS tests. The FT4, FT3, left STA-PSV, right STA-PSV, mean STA-PSV, 3 h and 24 h RAIU of the GD group were significantly higher than those of the thyroiditis group. The left STA-PSV, right STA-PSV and mean STA-PSV of the euthyroid group were significantly lower than those of the thyroiditis group (Table 1).

### Linear Correlation of Mean STA Peak Velocity and Iodine Uptake Rate

According to analysis, the mean STA-PSV and 3h RAIU (Fig. 1a, Y = 0.629X+42.36, *r* = 0.458, *P*<0.001) as well as 24 h RAIU (Fig. 1b, Y = 0.585X+35.57, *r* = 0.501, *P*<0.001) were significantly correlated in subjects with thyrotoxicosis (*n* = 304). Mean STA-PSV test was consistent with RAIU during thyrotoxicosis.

### Role of Mean STA-PSV in Differential Diagnosis of Thyrotoxicosis

The distribution of mean STA-PSV among subjects with euthyroidism, thyroiditis, and GD demonstrated a definite cutoff point between them (Fig. 2). In our retrospective study, the area under the ROC curve for mean STA-PSV in the differential diagnosis of thyrotoxicosis was 0.8799 (95% CI 0.8238–0.9359), while it was 0.9447 (95% CI 0.9110–0.9785) in the prospective study. According to the retrospective study, the optimal cutoff value of mean STA-PSV was 50.5 cm/s. We then applied this cutoff value (50.5 cm/s) in the prospective study and found that both the sensitivity and accuracy improved while the specificity remained unchanged (Table 2). Furthermore, we used this cutoff value of mean STA-PSV in another clinical center, where CDFS was performed with the same SOP through a different B ultrasound machine (GE LOGIQ 9 Color Doppler at Zhongda Hospital). As a result, both the sensitivity and specificity improved (Table 2). Our study validated that this test was worthy of promotion.

Moreover, comparisons were made between the diagnostic values of mean STA-PSV, TRab, and FT3/FT4 ratio in the etiological differentiation of thyrotoxicosis. As seen in Figure 3, the area under the ROC curves of mean STA-PSV and TRAb were comparable [0.901 (95% CI 0.839–0.963) vs. 0.902 (95% CI 0.839–0.964), respectively, *P*>0.05]. The differences were not statistically significant. However, both of them were markedly higher than that of FT3/FT4 [0.629 (95% CI 0.517–0.741), both *P*<0.001]. Mean STA-PSV and TRAb were comparable, regarding their values in the differential diagnosis of thyrotoxicosis.

### Analysis of Measurement Reproducibility

Table 3 details the coefficients of variation of STA-PSV detection. The coefficients of variation were lower than 10% for the euthyroid, thyroiditis and GD groups.

## Discussion

The etiological differentiation of thyrotoxicosis is commonly encountered in clinical practice. Sometimes it can be quite challenging, due to the possibility of painless thyroiditis and early-stage atypical GD [9]. Generally, the following methods are commonly used in the differentiation of transient thyrotoxicosis and GD: thyroid technetium^99^ scan, RAIU assay, T3/T4 ratio [10], [11], serum markers such as total activity of alkaline phosphatase [12] and TRAb [4], [13], [14]. Serological assays such as FT3/FT4 ratio [10], [11] and alkaline phosphatase activity had limited values in the differential diagnosis [10], [12]. Despite its application as a gold standard, RAIU can be easily influenced by diet and medications. TRAb assay is a commonly used diagnostic method for thyrotoxicosis differentiation in clinical practice. A large body of evidence shows that TRAb measured with 3rd generation immunoassay methods has very high sensitivity and specificity in the diagnosis of GD [15]. In China, CDFS plays an important role in the diagnosis of thyroid diseases. Almost all endocrinologists perform CDFS for patients with thyroid disease. CDFS is more accessible and available than RAIU and TRAb. In China, the patients do not have to wait for an appointment for CDFS, while most patients will have to wait for 1–2 weeks for an appointment for RAIU test. As indicated by our study, STA-PSV and TRAb had comparable values in differentiating thyroiditis and GD. However, up until now, there has been no literature report of the SOP for STA-PSV detection [16].

In the present study, SOP was established so that the operation discrepancies could be prevented. It is meaningful to compare the diagnostic values of mean STA-PSV in different clinical centers, only on the basis of a common detection SOP [1]. PSV detection at the intersecting point of STA and thyroid lobes has not been reported in previous studies. This unique detection point applied in our study is important for accurate positioning of STA. We chose STA rather than inferior thyroid artery (ITA). This is because STA is more superficial [4] and has fewer anatomic variations than ITA [7]. PSV is quantitative and reflects the status of thyroid blood supply. With the technology advance of modern color Doppler, PSV has become accurate and dependable, with an excellent reproducibility [16]. Compared with thyroid CDFS pattern judgment used in previous studies, STA-PSV detection is more objective and accurate [2]. As a result, our study has achieved an excellent reproducibility, which has been further confirmed by our reproducibility analysis among subjects with euthyroidism, thyroiditis and GD.

In both our retrospective and prospective studies, the cutoff point of mean STA-PSV at 50 cm/s had an excellent value in differentiating thyroiditis and GD. When different instrument from another manufacturer was applied, the diagnostic value of mean STA-PSV remained excellent, which validated that the PSV assay was worthy promotion. The good reproducibility of PSV assay may be attributable to the use of SOP and the dependability of color Doppler in measuring vascular velocity [17]. According to previous studies, the optimal STA-PSV cutoff point in differentiating thyroiditis and GD was in the range of 40–50 cm/s. A Japanese study obtained a PSV cutoff value of 42.5 cm/s from a sample size of 65 patients. By contrast, the cutoff value from our retrospective and prospective studies was about 50 cm/s. This discrepancy might be due to different sample size, constitution of study participants (inpatient/outpatient), measurement methodology (examination details were unknown in the Japanese study) and ethnicity. Compared with previous researches, the present study had a larger sample size. The data of both retrospective and prospective studies were included.

One interesting finding is that there are a few cases whose mean STA-PSV and TRAb were inconsistent. In the prospective study, we analyzed this discordance (Table 4). Among them, two cases had a fever due to subacute thyroiditis. We speculate that the blood flow velocity will increase during fever, which may also increase the mean STA-PSV to some extent.

In light of the consistency of the retrospective and prospective analysis, mean STA-PSV is proven to be practical, accurate and reliable in the differential diagnosis of thyrotoxicosis, especially when RAIU test is contraindicated during pregnancy and lactation.

## Supporting Information

Figure S1 Ultrasound image of subjects with euthyroidism.
(JPG)

Figure S2 Ultrasound image of subjects with Graves’ disease.
(JPG)

Figure S3 Ultrasound image of subjects with subacute thyroiditis.
(BMP)
