# Supplementary figures and images for: Peak Systolic Velocity of Superior Thyroid Artery for the Differential Diagnosis of Thyrotoxicosis
Source: PLoS One. 2012 Nov 16;7(11):e50051. doi: 10.1371/journal.pone.0050051 (PMC3500337; doi:10.1371/journal.pone.0050051)

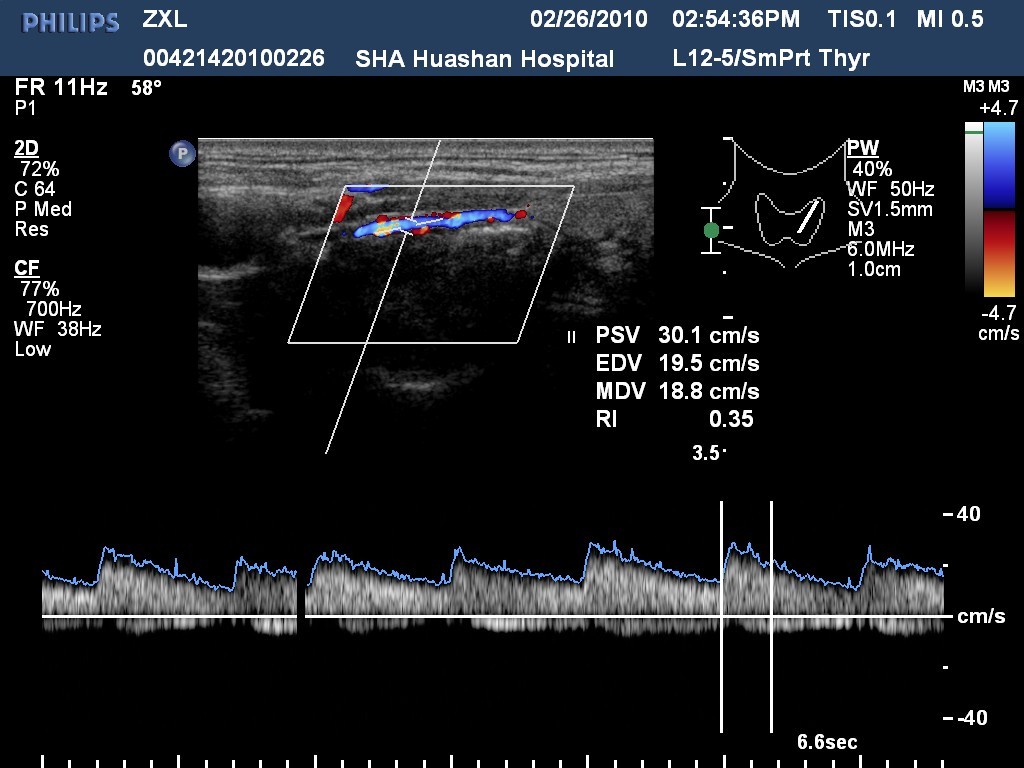

Supplement: Figure S1 — Ultrasound image of subjects with euthyroidism. (JPG) [file pone.0050051.s001.jpg]

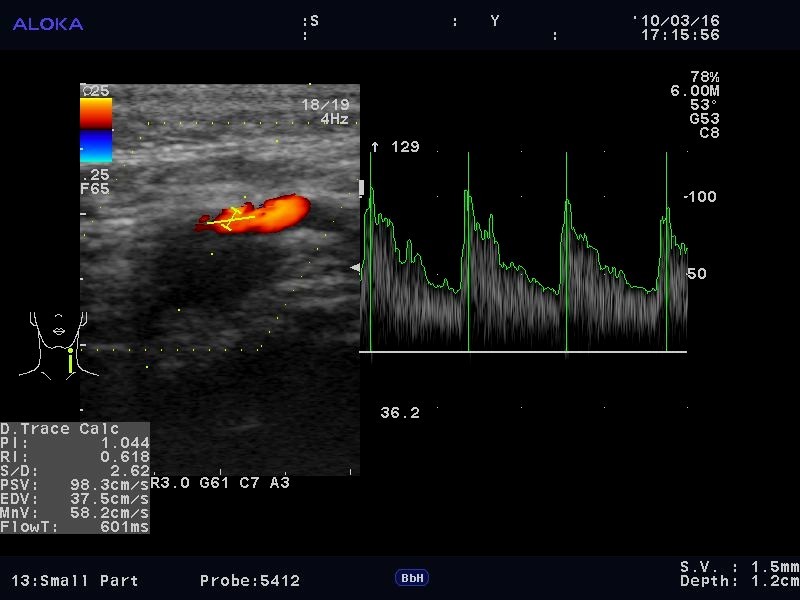

Supplement: Figure S2 — Ultrasound image of subjects with Graves’ disease. (JPG) [file pone.0050051.s002.jpg]

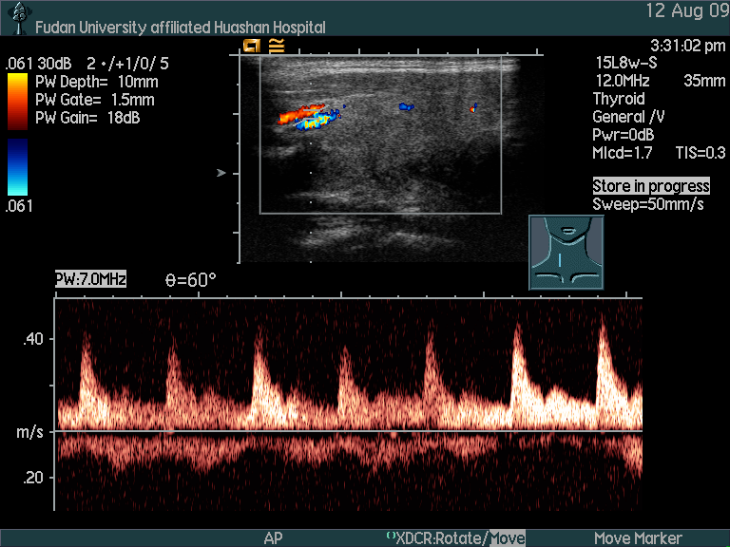

Supplement: Figure S3 — Ultrasound image of subjects with subacute thyroiditis. (BMP) [file pone.0050051.s003.bmp]
